# Hierarchical Image Transformation and Multi-Level Features for Anomaly Defect Detection

**DOI:** 10.3390/s23020988

**Published:** 2023-01-15

**Authors:** Isack Farady, Chia-Chen Kuo, Hui-Fuang Ng, Chih-Yang Lin

**Affiliations:** 1 Department of Electrical Engineering, Mercu Buana University, Jakarta 11650, Indonesia; 2 Department of Electrical and Communication Engineering, Yuan Ze University, Taoyuan 320, Taiwan; 3 National Center for High-Performance Computing, National Applied Research Laboratories, Hsinchu 300, Taiwan; 4 Department of Computer Science, University Tunku Abdul Rahman, Kampar 31900, Malaysia

**Keywords:** image transformation, poison image, feature vector, metal defect, anomaly detection

## Abstract

Anomalies are a set of samples that do not follow the normal behavior of the majority of data. In an industrial dataset, anomalies appear in a very small number of samples. Currently, deep learning-based models have achieved important advances in image anomaly detection. However, with general models, real-world application data consisting of non-ideal images, also known as poison images, become a challenge. When the work environment is not conducive to consistently acquiring a good or ideal sample, an additional adaptive learning model is needed. In this work, we design a potential methodology to tackle poison or non-ideal images that commonly appear in industrial production lines by enhancing the existing training data. We propose Hierarchical Image Transformation and Multi-level Features (HIT-MiLF) modules for an anomaly detection network to adapt to perturbances from novelties in testing images. This approach provides a hierarchical process for image transformation during pre-processing and explores the most efficient layer of extracted features from a CNN backbone. The model generates new transformations of training samples that simulate the non-ideal condition and learn the normality in high-dimensional features before applying a Gaussian mixture model to detect the anomalies from new data that it has never seen before. Our experimental results show that hierarchical transformation and multi-level feature exploration improve the baseline performance on industrial metal datasets.

## 1. Introduction

Anomalies are data that stand out amongst other data in a dataset and do not adhere to the normal behavior of the other data points. Anomaly detection thus refers to a process of detecting data that significantly lie outside of the majority data. The detection of anomalies and deviant patterns has been an active research area since various industries and business organizations strive to develop systems that are not only robust against deviant data, but can also detect them appropriately [1]. In prior decades, inspection methods for anomaly detection in industrial production lines mainly consisted of collecting images that experts would manually review for defects. Manual quality inspection is very inefficient in terms of time and labor for a company, but modern computer vision and deep learning techniques can address these issues. The drawback is that computer vision models with deep learning require a huge amount of data. However, the limitation in the industrial world is the availability of data: collecting more images is a particularly large challenge due to safety and security reasons. Moreover, in some cases, the percentage of anomalies in the dataset is extremely low, usually less than 1%. Since anomaly images are scarce and unknown to the user, researchers are seeking solutions for modeling the unsupervised normal or anomaly-free data distribution and defining a measurement in this normal data.

Deep learning-based models can be powerful tools for learning the features of training data and capturing the normal behavior of a normal data distribution. However, one major issue originating from an intrinsic attribute of DNNs is their ability to be affected by the input data. Because of their sensitivity to small perturbances, DNNs may be misled and misclassify images with a certain number of imperceptible perturbations [2]. As a result, when poison or non-ideal sample distributions are present in the testing data, features learned by the deep CNN may not be robust. Unfortunately, producing ideal samples is difficult in real-world data. As an example, in the steel production process, producing an ideal image is not easy due to the harsh work environment; moreover, capturing images in extremely high temperatures makes them very vulnerable to noise. In order to optimize the performance of anomaly detection models with poison samples, a strong and adaptive model is needed.

Today, deep learning-based anomaly image detection is used in many industries, including steel production. Currently, CNN methods such as VGG [3] and ResNet [4] are becoming de facto approaches for extracting the features for many anomaly detection problems [5,6,7]. A common way to implement deep learning-based models is to learn the feature representation of normality as presented in the survey paper [8], where the deep learning model is guided to search for the important features from normal data and defect data. Most of the models in the steel production industry use a technique to classify product defects, such as cracks, scratches, markings, missing parts, and inaccuracies in various inspection tasks [9,10]. These CNN-based models perform well when trained on vast amounts of ideal data. However, data subjected to noise, known as poison data, is a hindrance to the success of deep network-based anomaly detection systems in real-world applications. The original training data also do not cover the full range of possible future anomaly or defect classes. Within this reality, data scarcity is not the only constraint we face; it is also crucial to obtain additional ideal representative images.

Ideal or uniform samples can be defined as similar data variations that typically share the same conditions, such as image quality, exposure, and lighting conditions. Currently, the existing methods [11] for industrial anomaly detection are generally suitable for uniform images that possess the ideal conditions. However, in practice, the distribution of testing images from industrial image collections can vary in terms of quality and conditions. In this situation, the testing samples may include random quality of samples that obstruct the success of current models.

This work mainly focuses on anomaly detection in an industrial manufacturing inspection context. To address non-uniform data in this domain, we design a novel additional module that explores the benefit of robust image transformations to introduce variation into existing normal images. At the same time, a combination of multi-level features is added to a multivariate Gaussian distribution model to enhance the normality learning process. Our proposed method, Hierarchical Image Transformation and Multi-level Features (HIT-MiLF), explores the new additional transformation samples and improves the relationship of high-dimensional features of CNN. Our approach can be viewed as an additional module to assist in the feature extraction process for non-ideal or poison images. Unlike other works, our method exploits the model to learn more variety from normal images rather than introducing variety into normal images for anomaly detection. We prove that the model becomes more sensitive to perturbances in testing samples for both normal and defect samples. Our method also achieved higher prediction scores on test images with various poison levels compared to a model without HIT-MiLF.

In summary, our contributions are as follows:

- We introduce a novel hierarchical transformation module for anomaly detection. With this approach, the anomaly detection model not only contains robust normal data but also becomes more resistant to poison and non-ideal image variation.
- We introduce a method that combines the hierarchical transformation process and multi-level feature selection for anomaly defect detection. Our method can easily be extended to the few-shot or zero-shot anomaly detection problem.
- We demonstrate consistent gains in testing on several non-ideal image simulations and exceed the baseline performance.

Our paper is organized as follows. In Section 2, we give an overview of other works and corresponding methods. In Section 3, we present our method, provide a comprehensive workflow of hierarchical concepts, and discuss the multi-level feature process. We then analyze the experimental results on the Metal Casting (MC) dataset and MVTec Metal-nut dataset in Section 4. We finish with our conclusions in Section 5.

## 2. Related Work

In this section, we primarily discuss relevant work on anomaly defect detection with deep learning-based approaches, image transformation, and feature representation of normality and industrial anomaly detection. In recent years, deep learning-based models have shown tremendous capabilities in learning expressive representations of complex data, such as high-dimensional data, sequential data, and image data. Based on the handling of data variations, anomaly detection approaches can be classified into distribution-based methods and reconstruction-based methods.

### 2.1. Distribution-Based Methods

There is a tendency for anomalous data to fall into low-probability regions that are distributed throughout the normal data. In this scenario, distribution-based methods try to predict whether the new sample lies in a high-probability region or not. The most straightforward version of anomaly detection uses a simple statistical approach, wherein statistical techniques such as mean, median, and quantiles can be used to detect univariate feature values in the dataset [12]. However, simple statistical rules are prone to producing more false negatives and false positives. Conventional distribution-based methods for anomaly detection, such as SVM [13,14], one-class SVM [15], and kernel density estimation [16,17,18] are fragile when dealing with high-dimensionality data. The drawbacks of distribution-based techniques spawned a considerably more robust method using deep learning for anomaly detection. A deep learning model trained on anomaly detection is a classic yet challenging task that has numerous use cases across various domains, such as fraud detection [19,20,21], cyber security [22,23], time series analysis [24], and medical applications [25,26,27]. The challenge in anomaly detection with deep learning comes mainly from the fact that the task is data-scarce by definition.

### 2.2. Reconstruction-Based Methods

Anomaly detection approaches can also be classified as reconstruction-based. In this method, autoencoders can learn shared patterns of normal images and restore them correctly. In [28,29], the models estimate pixel-level reconstruction errors as anomaly scores. PCA-based [30] and autoencoder-based methods [31] rely on the perceptual loss, where the models trained only on normal data cannot accurately reconstruct anomalies. Apart from autoencoders, recent models [32,33,34] have used a GAN-based architecture as a detection method. In GAN-based anomaly detection models, GAN is applied to generate samples from scratch according to training data. Given test data, GAN-based models try to find the point in a generator’s latent space that generates the sample closest to the considered input. Intuitively, if the GAN is able to capture a good representation of the test image, then the image is normal, and vice versa. Other generative models [35] learn distributions of anomaly-free data and estimate the reconstruction error metrics for unseen images with anomalies. Similar to autoencoders, a major difficulty with generative-based models lies in how to regularize the generator for compactness [36,37,38].

### 2.3. State-of-the-Art Anomaly Detection

Numerous deep learning-based methods have emerged in anomaly detection as discussed in this survey [8,11,23,39]. In industrial domains, research on big data presented in [40] proposed a variational long short-term memory (LSTM) learning model for anomaly detection on reconstructed feature representation. A variation of a self-supervised pretrained model, Patch SVDD [41] proposed combining multi-scale scoring masks to the final anomaly map. In [42], the proposed deep invertible network showed that large feature representation from ImageNet [43] can be more representative for the pretrained model to compare to a small specific dataset e.g., the industrial public MVTec dataset [44], or a medical image dataset [45,46]. Adopting the benefit of a huge ImageNet pre-trained model, PaDIM [47] proposed patch distribution modeling, which uses patch embedding from pretrained CNN and captures the probability with a multivariate gaussian distribution. In order to estimate the features vector of each sample from pooled feature maps [48] and some other popular models [49] use the Mahalanobis distance metric [50,51]. Another GAN-based anomaly detection method has become a popular deep learning anomaly detection approach since its introduction in [52]. This approach generally aims to learn abnormal inferences using adversarial learning of the representative of samples [53,54]. GAN-based models in anomaly detection are designed for reconstruction-based methods, where, in general terms, the simplest approach is to take the benefit of the reconstructed error as an anomaly score [55].

Inspired by these state-of-the-art anomaly detection works, we aim to explore the variations of normal images before distributing the new samples into the feature extractor. Image transformation in anomaly detection has been presented in [56], where geometric transformation is implemented to discriminate between many types of transformation and normal images to detect anomaly samples. Similarly, the latest geometric transformation in [57] was designed for few-shot learning. In contrast, HIT-MiLF utilizes the image generator to produce new normal samples from a pixel-wise transformation in batches and keeps the original label for new samples. In this way, we can say that HIT-MiLF is not costly in terms of labeling.

## 3. Method

In this section, we discuss the anomaly detection settings followed by the combination of hierarchical transformation and multi-level features. These modules are part of the data enhancement for the CNN to learn the invariance of normal training data. We first describe the anomaly detection setting that covers the whole structure of this work in Section 3.1. We explain the hierarchical transformation module that we add to the anomaly detection model in Section 3.2. We then explain Multi-level Features (MiLF) in Section 3.3, and end by discussing multi-level feature representation of the new generated samples.

### 3.1. Anomaly Detection Setting

**Figure 1:**
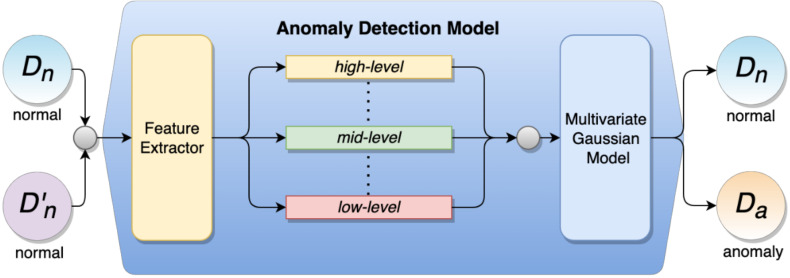
Hierarchical image transformation (HIT) module and multi-level features (MiLF) added to an anomaly detection model. *D_n_* represents input training data from a one-class normal image; the output of the model is normal (*D_n_*) or anomaly (*D_a_*).

In this paper, we consider the problem of anomaly detection, specifically in industrial images. Given a dataset D, the deep anomaly detection model aims to learn the feature representation mapping function $𝓕:D_{x}\to D_{y}$ where $D_{x}$ is training data (one-class normal sample) and $D_{y}$ is the output prediction (Figure 1). In the testing phase, the predicted samples $D_{y}$ can be represented as $D_{y}=\left\{D_{n}\;\vee \;D_{a}\right\}$ where $D_{y}$ contains either normal data $D_{n}$ or anomaly data $D_{a}$. We adopt ResNet18 [4] as the backbone of our network, which extracts the feature of $D_{n}$ before exploring the feature vector from a different block.

In accordance with typical anomaly detection settings, we train a network with a given sample of all normal images $D_{n}$. In an ideal condition, $D_{n}$ is trained with network M to capture high-dimensional feature vectors. In anomaly detection, the anomaly-free data distribution is commonly estimated using multivariate Gaussian distribution N(μ,Σ), where μ is the mean and Σ is the covariance. We follow PaDIM [47] to learn the anomaly-free samples at a specific patch position (i,j), and learn the normality from the set patch embedding vector at (i,j). At the specific (i,j) position, $X_{ij}=\left\{x_{ij}^{k},k\in \left[1,\;D_{n}\right]\right\}$ from n normal images and the multivariate Gaussian distribution $N\left(\mu_{ij},\Sigma_{ij}\right)$. The covariance of normality characterization at (i,j)  position is estimated as follows:

(1) $$\Sigma_{ij}=\frac{1}{D_{n}}\overset{D_{n}}{\underset{k=1}{\sum }}\left(x_{ij}^{k}-\mu_{ij}\right){\left(x_{ij}^{k}-\mu_{ij}\right)}^{T}+\epsilon I$$

The regularization ϵI makes the sample covariance matrix $\Sigma_{ij}$ invertible. Finally, each (i,j) patch position is associated with multivariate Gaussian parameters.

### 3.2. Hierarchical Transformation

The anomaly classification system should be trained with as many variations of the considered objects as possible. One major problem lies in industrial dataset availability: real public defect images are difficult to obtain, since anomalous images are extremely rare and sometimes, the defects in production lines involve sensitive data that is not easy to access. In [8], the authors presented a publicly available real-world public dataset. Although publicly accessible datasets are available, most of them contain sequential data. In this work, we mainly focus on industrial images and implement our approach on this type of data.

Anomaly defect detection faces a challenge where the training data contain only one-class normal data. Our proposed HIT module consists of two main parts: the sample generator and the sample collector. These parts are assembled into one module to process all possible normal data. The output of this module is distributed to the CNN via multiple training batches.

Let $T=\left\{{Τ}_{1},\;{Τ}_{2},{Τ}_{3},\ldots ,{Τ}_{n}\right\}$ be a set of pixel transformations where ${Τ}_{n}:\;D\to \;D_{T\left(n\right)}^{\prime }$ and $D_{n}$ is the initial or identity samples. The set of T in the image generator is based on the intuition of pixel-level transformations properties on anomaly detection. Pixel-level transformations keep the spatial structures and maintain the detailed artifacts of normal samples. In this work, transformation T includes hue saturation, noise injection, adding shadow effect, and adjusting brightness and contrast. In the first iteration of the hierarchical process, the original image $D_{n}$ will directly distribute to bacth_1 without any transformation. The next iteration, the image of $D_{n}$ will be processed in the transformation module ${Τ}_{1}$ and produce the new transformed sample of a normal image $D_{T1}^{\prime }$ This hierarchical process will repeat according to the available set of T where we apply the set of T, for all original samples to the generator. The generator with ${Τ}_{n+1}$ will generate another new samples of $D_{T\left(n+1\right)}^{\prime }$ from the combination of $D_{n}$ and $D_{T\left(n-1\right)}^{\prime }$ to the next iteration.

**Figure 2:**
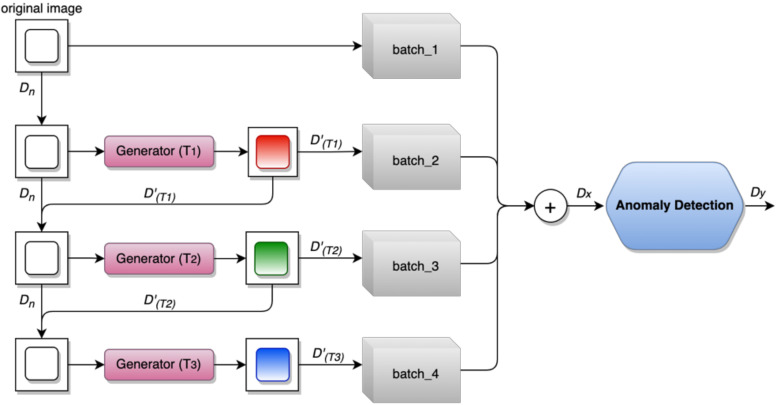
Hierarchical image transformation flows in the data processing for anomaly detection network. The transformations keep the transformed samples class as the original *D.* New samples (red, green, and blue) represent the new transformation of the original $D_{n}$ from different transformations process.

As shown in Figure 2, the class of new samples $D_{T\left(n\right)}^{\prime }$ will be the same as the normal image after transformation. Here, the new samples represent the normal image in different pixel-level conditions. This modification is what we want to achieve through this approach: we assume that the diversity of images from the original will unlock more informative features that represent the anomaly-free data. The new samples $D_{T\left(n\right)}^{\prime }$ will be distributed in multiple batches and directly forwarded to the CNN as new input training data.

### 3.3. Learning Multi-Level Feature Representation

**Figure 3:**
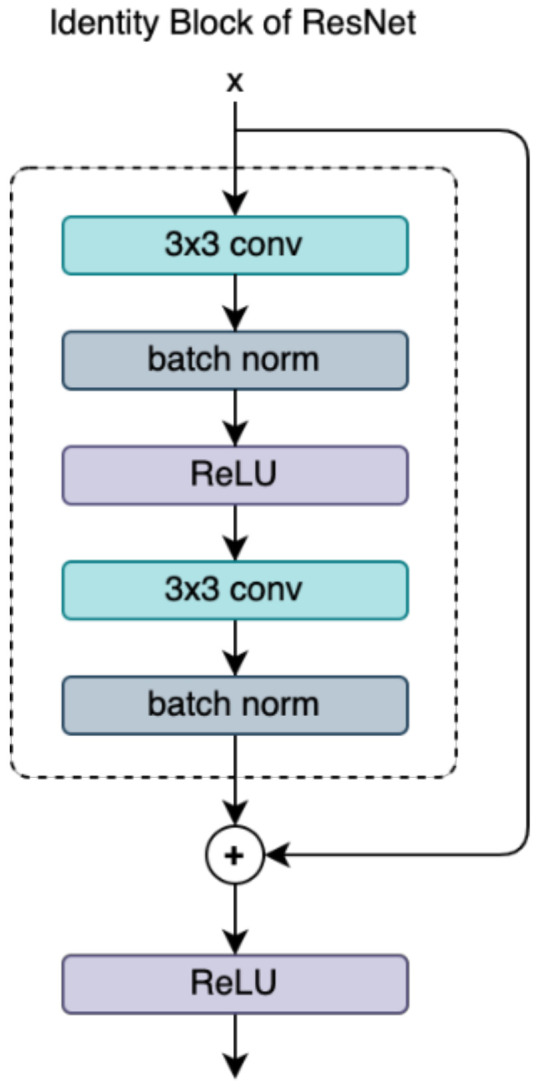
Identity residual block of ResNet18.

**Figure 4:**
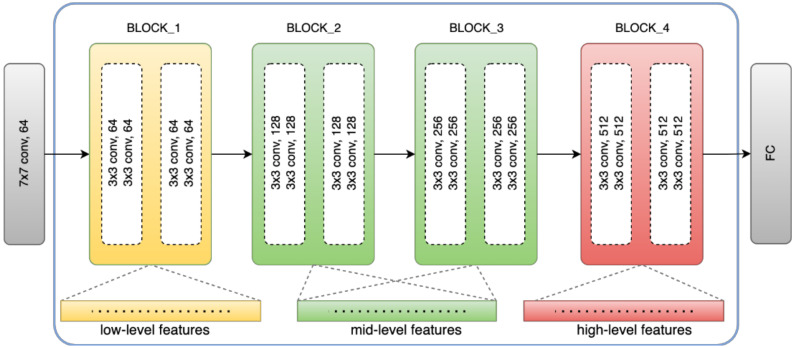
Multi-level feature representation from three different blocks of ResNet18.

Compared with traditional feature extraction methods, the CNN-based feature extractions are more capable of extracting feature distribution information. Moreover, this ability to extract high-level semantic information enables the model to be trained end-to-end. Currently, various backbone networks have been used in previous work, such as [37,38,50], etc. In this work, we adopt ResNet18 to capture high-dimensional features of input training images. The details of the ResNet18 block structure are presented in Figure 3 and Table 1. As shown in Figure 4, the backbone includes four blocks, and these blocks extract appearance information from the low-level (block_1) and middle-level (block_2 and block_3) to the high-level (block_4). With the exception of the last block, each block consists of convolution levels, a rectified linear unit activation function (ReLU), batch normalization, and a max-pooling layer. These different blocks are fused by leading input and posterior output features to enrich the feature map. Since every feature output of this block can be retrieved as a high-dimensional feature vector, we explore this advantage to collect all feature outputs from each block.

However, in the complex condition of normal samples, high-dimensional features from a deep neural network cannot fully describe the normality feature of training data. This is because there is a lack of variation among limited anomaly-free training data. Therefore, it is crucial to enlarge the data in order to enrich the variation and strengthen the data complexity. To address this issue, apart from generating new samples in the single HIT module, we propose a combined model that jointly uses HIT and multi-level features from ResNet18 to extract variations of anomaly-free images. We utilize the multi-level features from different blocks of ResNet18 to capture the different relationship and semantic information features from normal samples.

As shown in Figure 4, we collect the extracted features from specific blocks and concatenate the activation vectors. The idea behind this approach is that the typical deep convolutional layer property of CNN, or different layers of deep CNN, can encode different levels and shapes of information. Low-layer features always contain more detailed information and have higher resolution. In other words, the first block of CNN contains features encoded with less context. However, in high-level blocks, the features encode more contextual or semantic information in low spatial resolution. Directly combining the low-level and high-level features may affect the feature concatenation that causes semantic ambiguity due to the introduction of high-detailed information. To address this concern, we exploit the middle level that acts as an intermediary feature representation between the low level and high level in order to bring about transition information. We show the effect of the feature-level block selection on the final anomaly prediction in Section 4.3.

After multi-level feature concatenation, the embedding vectors carry information from different semantic levels. We estimate the multivariate Gaussian distribution of N(μ,Σ) of the feature vectors from three levels. In this model, we partition the input image into patches and calculate the distribution before the multivariate distribution. We distribute all combination features provided by MiLF to ensure both images $D_{n}$ and $D_{T\left(n\right)}^{\prime }$ are treated consistently.

## 4. Experiments

### 4.1. Anomaly Detection Setting

**Figure 5:**
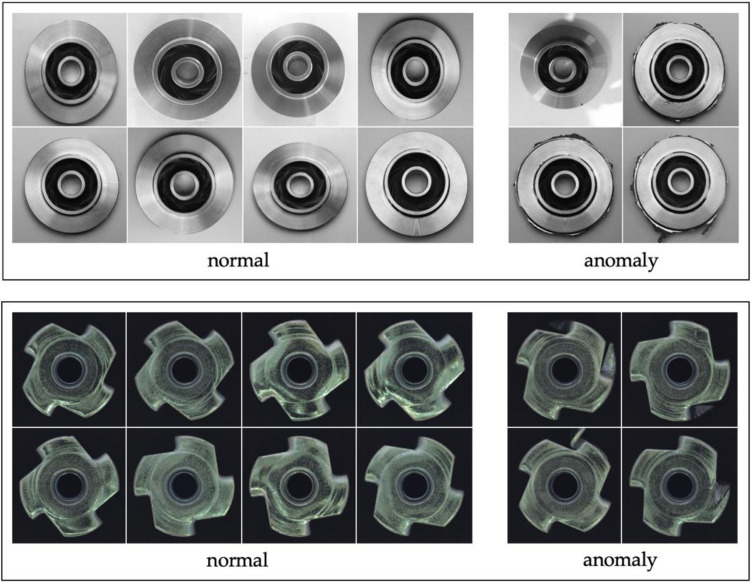
Sample images from MC dataset (**first row**) and MVTec Metal-nut dataset (**second row**).

Dataset. In this experiment, we use the Metal Casting dataset [58] and Metal-nut from MVTec dataset [44] to detect anomalous defects for vision quality inspection. Metal casting is a manufacturing process in which a material is poured into a mold that contains a hollow cavity of the desired shape. There are many types of defects in metal images, such as blow holes, mold material defects, shrinkage defects, pinholes, scratch, etc. However, the objective of this work is mainly to detect anomalous image from only available normal images. The original MC dataset contains 1300 images of 512 × 512 pixels with 781 defect images and 519 non-defect images. The Metal-nut dataset consists of 335 images with 242 non-defect images and 93 defect images. In preparation for examining the model, for MC dataset, we use the 500 of 519 images and split into 400 non-defect images for training and 100 images for validation. We select 100 of 781 defect images in testing for five different poison levels. In this setting, one poison level contains 400 non-defect for training and 100 non-defect and 100 defect images for testing. For the Metal-nut dataset, we apply 220 non-defect images for training. For the testing set, we use 22 non-defect and 93 defect samples to perform poison levels test. The sample images from our datasets are shown in Figure 5.

Metrics. In a common classification model, accuracy is an acceptable metric that measures the number of predictions that are correct as a percentage of the total number of predictions. Accuracy as a prediction metric is suitable only when an equal distribution of classes exists in the testing set. However, in anomaly detection, we need to control the sensitivity of the model because the model may classify all testing data as anomalous, even though they are not (false positive). Thus, in the field of anomaly detection, the most suitable metrics that have been used in many works are F1 score and area under the curve (AUC) score. The F1 score is defined as the harmonic mean of precision and sensitivity or recall and is often useful when computing an average rate. The formula for the F1 score is as follows:

(2) $$\mathrm{Precision}=\frac{\mathrm{True}\;\mathrm{Positive}}{\mathrm{True}\;\mathrm{Positive}+\mathrm{False}\;\mathrm{Positive}}$$

(3) $$\mathrm{Recall}=\frac{\mathrm{True}\;\mathrm{Positive}}{\mathrm{True}\;\mathrm{Positive}+\mathrm{False}\;\mathrm{Negative}}$$

(4) $$F1\;\mathrm{score}=2*\frac{\mathrm{Precision}*\mathrm{Recall}}{\mathrm{Precision}+\mathrm{Recall}}$$

**Figure 6:**
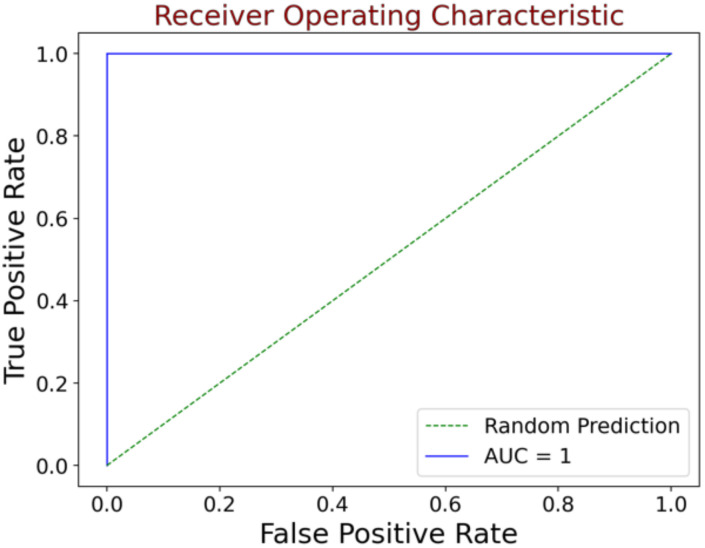
The illustration of the ideal AUC score = 1 where false positive rate is zero and true positive rate is one. This means that a larger area under the curve is better.

The AUC score is the second metric in the field of anomaly detection where it measures the area underneath the receiver operating characteristic (ROC) curve. The AUC is a probability curve that plots the true positive rate (TPR) against false positive rate (FPR) at various threshold values. AUC integrates the classification performance between the normal image and defect image for all decision thresholds. Since the AUC represents the degree or measure of separability, it is suitable as a performance measurement in various settings. This metric indicates how well a model can distinguish between two classes. AUC ranges in value from 0 to 1, where the higher the AUC score, the better the model is at predicting normal and anomaly. The illustration of the perfect AUC score is shown in Figure 6.

(5) $$\mathrm{True}\;\mathrm{Positive}\;\mathrm{Rate}=\frac{\mathrm{True}\;\mathrm{Positive}}{\mathrm{True}\;\mathrm{Positive}+\mathrm{False}\;\mathrm{Negative}}$$

(6) $$\mathrm{False}\;\mathrm{Positive}\;\mathrm{Rate}=\frac{\;\mathrm{False}\;\mathrm{Positive}}{\mathrm{False}\;\mathrm{Positive}+\mathrm{True}\;\mathrm{Negative}}$$

### 4.2. Implementation Details

We conducted an anomaly detection experiment using two methods: an ideal or uniform sample test, and poison and non-ideal sample test. We defined our baseline as a standard anomaly detection model that uses the ideal training and poison-free testing data. The result of our baseline is presented in Table 2. We ran our models on a computer with a single Nvidia 1080i GPU card and used a PyTorch-based framework [59]. As shown in Table 2, the AUC metric of the anomaly detection baseline reached 0.973 (AUC score), 0.917 (F1 score) and 0.934 (AUC score), 0.928 (F1 score) on the MC dataset and Metal casting dataset respectively. Similar to the baseline model, we used our model on an ideal image and produced similar scores, which indicates that although our model was designed for non-ideal data, it can still be used on ideal data with an acceptable level of accuracy that is competitive with the original model.

We then simulate the poison sample test images to validate our proposed method on poison and non-ideal images, for instance, with noise injection, blurring, and image sharpening. In this setting, the testing data contains poison and non-ideal samples that randomly set the number of samples for both normal and anomaly classes. We rank five levels of poison samples in testing data, from Level 1 to Level 5 which the number of poison images at each level increases by 10% relative to the original testing data. We re-run our baseline with this approach to show how poison and non-ideal samples severely weaken the baseline.

### 4.3. Results

#### 4.3.1. Experiment with HIT Module

In the first experiment, we assessed and compared the effectiveness of single HIT module with poison and non-ideal images on MC dataset and Metal-nut dataset. From the experimental results in Table 3, we observed that our baseline results dropped significantly when poison and non-ideal testing data were added. This phenomenon also occurs in several subsequent experiments with an increasing number of poison and non-ideal samples across two datasets. This indicates that the baseline model is confused by the new poison and non-ideal data, which are comparatively different from the ideal data. The poison images cause the model to fail to retain the informative features of normal images. To work around this issue, we then attach the HIT module to the baseline model while we maintain all settings and the detailed structure of our CNN model. The main goal of the HIT module is to generate new additional training samples as a means of enhancing the CNN to automatically extract more informative features from different image transformations.

Table 3 presents our experimental results on poison and non-ideal testing data for both the baseline and the baseline with the additional HIT module on MC and Metal-nut dataset. We analyze the results for every percentage level of poisoning testing data and plot the metric scores for all levels of poison samples. At all levels of poison samples, we observe that both the AUC score and F1 scores gradually drop. This phenomenon appears not only in the baseline results but also in our HIT module. However, the baseline score strikingly jumps for AUC at Level 1 (10% poison samples). In contrast with our methods, the HIT model experienced a decrease of 0.05 and at the same level on MC dataset. We hypothesize that poison samples strongly affect whole feature distribution. This condition indicates that when a large number of poison samples appear in testing data, the robustness of the model significantly decreases. On the other hand, even though HIT experienced a similar weakening situation, it maintained a competitive score and consistently performed above the baseline. In Table 3, we also noticed that the F1 score in our single HIT module for MC dataset was slightly low at the first increasing poison sample. We assume that the HIT module is not sufficiently stable with a small number of poison images.

#### 4.3.2. Experiment with the MiLF Module

In the second experiment, we investigated the influence of the multi-level features on our backbone, ResNet18, and analyzed the model’s performance for every poison and non-ideal level of testing data. The results in Table 4 show that both the AUC score and F1 score consistently meet or outperform the baseline. Notably, the average AUC score stays above 0.9 across all poison data levels for MC dataset. We observed that a larger number of poison images affects all metrics and methods gradually, but that our proposed method still outperforms the previous method.

During the experiments, we inspected the layer by automatically selecting the best layer combination of the features level. From this experiment, we learned that multi-level implementation is needed to give a better feature representation of normality to adapt to poison samples. The experimental results in Table 4 show the score when multi-level features are included. We utilized the same combination of three main levels of feature representation (low-level, middle-level, and high-level). However, using the highest feature level from block_4 was not always the best option in this case, as doing so resulted in a low prediction score compared to other lower-level features from block_1. The benefit of the MiLF method lies in how we can combine the characteristics of high-resolution information from the high-level (block_4) with basic information from the low-level (block_1) features, where the semantic level information can help improve the accuracy.

Additionally, we explored the benefit of features-levels from different blocks to investigate the effect of implementing several blocks of ResNet18 on MC and Metal-nut datasets. We run the experiment in the normal setting of anomaly detection where all features from different levels of ResNet18 were collected before feed into anomaly model. In this experiment we manually selected the features-level from ResNet18 blocks and followed anomaly detection model for each level. First, we performed the model on high-level (block_4) then combined with other lower blocks. As shown in Table 5, combining the different levels yields higher result than only using high-level features. This result inline with MiLF concept that we want to explore the optimum level combination from deep feature extractor.

#### 4.3.3. Experimental Results with the HIT and MiLF Combined

**Figure 7:**
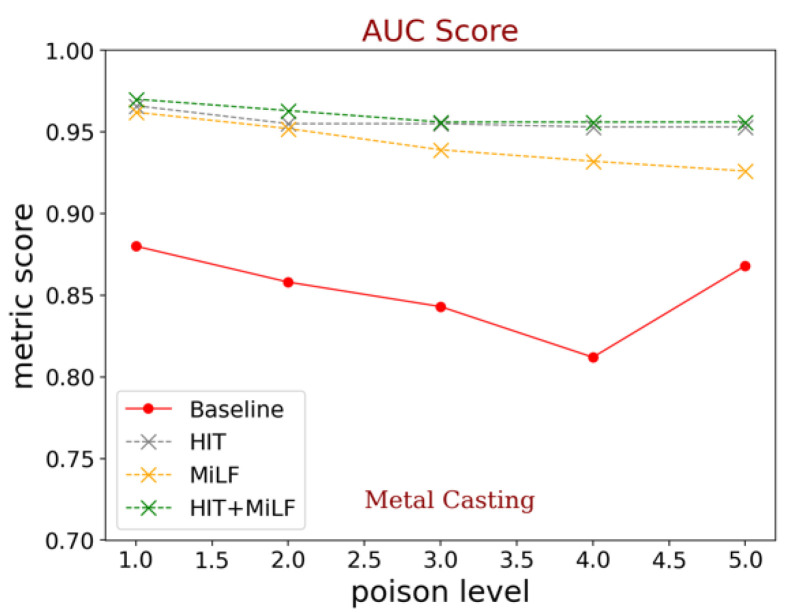

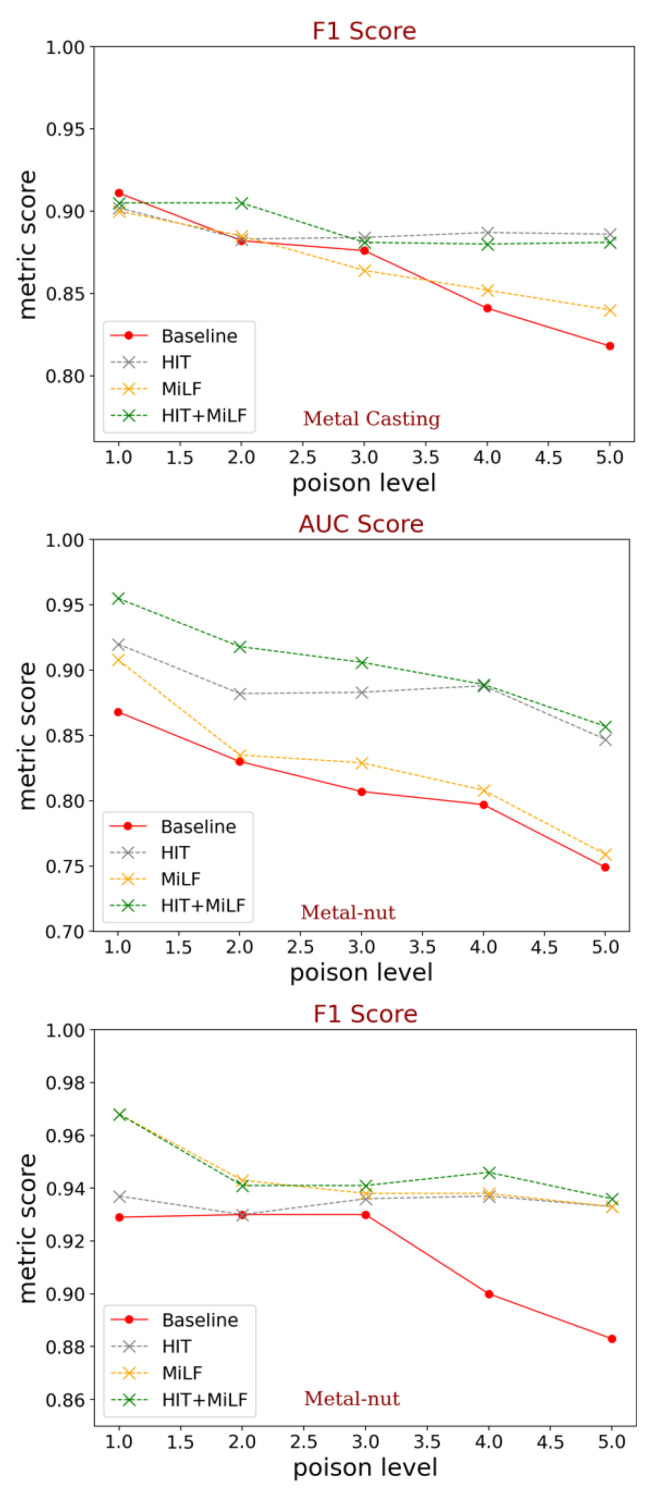
Comparison results of the model before and after implementing our proposed idea across various levels of poison sample on testing data. Although the trend line decreases as poison data increases (up to 50%), our proposed model is still more resistant against poison data and more competitive than baseline models as shown in Metal Casting and Metal-nut subfigures.

In the third experiment, we extended our ideas to demonstrate the effectiveness of our proposed models by combining the HIT module and MiLF into a single process and discussing several implicit factors that can influence anomaly detection. In the previous experiment (Section 4.3.1.), we briefly discussed what kind of transformations we can apply in the HIT module. In the first stage, we prepared the HIT module to produce new transformation samples and store the new sample in batches before distribution to the CNN. To perform multi-level feature selection, we used the same ResNet18 structure as in previous experiments, where the basic difference was the number of training data after adding the HIT samples. To inspect the variation of new samples, we ran the HIT + MiLF structure with ResNet18 in five different combinations according to the scalability of poison images. ResNet18 extracted the features of input images, including original samples, and generated new samples from each batch. Inside the feature extractor, we successively collected the extracted features from three different levels (low-middle-high level), where we used the highest validation score of each level before selecting a specific layer. The combination of these three layers returned better performance with this approach. As shown in Figure 7, combining HIT and multiple layers in MiLF outperformed the baseline on all poison levels. These results indicate that the new transformed samples and the optimum multi-level features can effectively improve normality learning from high-dimensional normal features. From these results, we notice that selecting the feature level from a single block of ResNet18 does not change the results significantly. However, the major effect of the combination multi-level model is to make the features from different levels uniform in resolution and dimensionality.

We present the comparison results from all of our experiments in Figure 7. As shown in Figure 7, the red line represents our baseline scores, the gray is the baseline with the HIT module, the orange is the baseline model with MiLF, and the green line represents the combination module. The results clearly show that each proposed model basically suffers from a decrease in performance as the poison data increases. However, what we perceive from these experiments is that the diversity of normal images makes the features more diverse, and that it is useful to capture the anomalies from outlier distribution samples. This phenomenon leverages the final distribution mapping on Gaussian models; as a result, our combination model is resistant to poison data and can maintain competitive results. Overall, these experiments prove that our proposed model can handle not only high-level features, but poison-free images as well. An additional interesting aspect of our proposed idea is that the combined structure is relatively light and easy to implement for all ResNet variants.

In Table 6, we show the experiment results for anomaly detection on MC dataset and Metal-nut dataset from various existing anomaly detection methods. We reimplement the same ResNet18 backbone as feature extractor to compare the benefit of adopting hierarchical transformation and multi-level feature combination. We notice that Probabilistic modeling-based outperforms our approach at low level of poison samples. However, the existing methods drop the score as the number of poison samples increases. The results presented in Table 6 show that our combination approach is relatively consistent across two popular modeling-based models for all poison levels. This confirms that hierarchical image transformations and multi-level layer selection approach for feature selection is crucial to handle poison samples in anomaly detection.

#### 4.3.4. Limitations

Here, we want to discuss some limitations of this approach based on our experiments. Since the HIT module is designed to produce new samples in a hierarchical way, it causes an increasing number of iterations and consumes more time. However, it is heavily dependent on the number of original images. In a scenario where normal training images are extremely limited, the search process will easily stagnate when searching the variations. We found that HIT baselines with various transformation methods are easily saturated when the original images are extremely limited.

## 5. Conclusions

In this empirical study, we have seen that informative features of normality create a strong foundation for an anomaly detection model to detect anomaly samples. Robust training data are important for teaching an anomaly model to be more sensitive to any perturbances from unseen samples. With hierarchical transformation samples, the CNN backbone is able to extract more informative features that surprisingly produce better features. From the multi-level feature combination, we can observe that combining lower- and high-level features with the help of the middle level can produce very competitive scores compared to a non-hierarchical model. This leads to the conclusion that a fully trained model combining hierarchical and multi-level features can push the model toward random poison images. Since this approach is light, our proposed model can be implemented on production lines.

Future work. Image transformation and multi-level feature combination pave the way to numerous extensions. In future work, we will study the application of our approach to other domains, e.g., medical images, natural images, or non-industrial datasets, where the anomalous data scarcity remains the bottleneck.

## Figures and Tables

**Table 1:** ResNet18 network structure.

Block_ID	Layer	Output Size	Configuration	
	conv1	112 × 112 × 64	7 × 7 × 64, stride 2	
Block_1	conv2_x	56 × 56 × 64	3 × 3 max pooling, stride 2	
			[3 × 3 × 64]	×2
			[3 × 3 × 64]	
Block_2	conv3_x	28 × 28 × 128	[3 × 3 × 128]	×2
			[3 × 3 × 128]	
Block_3	conv4_x	14 × 14 × 256	[3 × 3 × 256]	×2
			[3 × 3 × 256]	
Block_4	conv5_x	7 × 7 × 512	[3 × 3 × 512]	×2
			[3 × 3 × 512]	
	average pooling	1 × 1 × 512	7 × 7 average pooling	
	fully connected	1000	512 × 1000 fully connections	

**Table 2:** Baseline results on ideal testing data.

Dataset	Metric	Baseline	HIT	HIT + MiLF
Metal casting	AUC/F1Score	0.973/0.917	0.967/0.917	0.972/0.915
Metal-nut	AUC/F1Score	0.934/0.928	0.935/0.933	0.957/0.950

**Table 3:** Comparison of results between the baseline network with and without the addition of our proposed HIT module on five levels of poison samples.

(a) MC Dataset	AUC Score		F1 Score	
Poison Samples	Baseline	HIT	Baseline	HIT
Level-1 (10%)	0.880	0.966	0.911	0.902
Level-2 (20%)	0.858	0.955	0.882	0.883
Level-3 (30%)	0.843	0.955	0.876	0.884
Level-4 (40%)	0.812	0.953	0.841	0.887
Level-5 (50%)	0.868	0.953	0.818	0.886
Average	0.852	0.956	0.866	0.888
(b) Metal-nut dataset	AUC Score		F1 Score	
Poison Samples	Baseline	HIT	Baseline	HIT
Level-1 (10%)	0.868	0.920	0.929	0.937
Level-2 (20%)	0.830	0.882	0.930	0.930
Level-3 (30%)	0.807	0.883	0.930	0.936
Level-4 (40%)	0.797	0.888	0.90	0.937
Level-5 (50%)	0.749	0.847	0.833	0.933
Average	0.810	0.884	0.904	0.934

**Table 4:** Comparison results between the baseline and the baseline combined with our proposed HIT methods, as tested across various percentages of poison samples in testing data.

(a) MC Dataset	AUC Score		F1 Score	
Poison Samples	Baseline	MiLF	Baseline	MiLF
Level-1 (10%)	0.880	0.962	0.911	0.900
Level-2 (20%)	0.858	0.952	0.882	0.885
Level-3 (30%)	0.843	0.939	0.876	0.864
Level-4 (40%)	0.812	0.932	0.841	0.852
Level-5 (50%)	0.868	0.926	0.818	0.840
Average	0.8522	0.9422	0.8656	0.8682
(b) Metal-nut dataset	AUC Score		F1 Score	
Poison Samples	Baseline	MiLF	Baseline	MiLF
Level-1 (10%)	0.868	0.908	0.929	0.968
Level-2 (20%)	0.830	0.835	0.930	0.943
Level-3 (30%)	0.807	0.829	0.930	0.938
Level-4 (40%)	0.797	0.808	0.90	0.938
Level-5 (50%)	0.749	0.759	0.833	0.933
Average	0.810	0.827	0.904	0.944

**Table 5:** Comparison results between different features-level selection for MiLF module.

	MC Dataset		Metal-Nut Dataset	
Level Features	AUC Score	F1 Score	AUC Score	F1 Score
High	0.937	0.917	0.934	0.928
Low + High	0.951	0.883	0.951	0.947
Middle + High	0.947	0.894	0.941	0.937
Low + Middle + High	0.945	0.897	0.985	0.978

**Table 6:** Comparison results with other deep features-based methods on Metal casting and Metal-nut datasets.

(a) Metal Casting	AUC Score				F1 Score			
Poison Samples	PaDIM [47]	KDE Modeling-based	Probabilistic Modeling-based [60]	HIT + MiLF	PaDIM [47]	KDE Modeling-based	Probabilistic Modeling-based [60]	HIT + MiLF
Level-1 (10%)	0.881	0.902	0.930	0.97	0.874	0.920	0.947	0.905
Level-2 (20%)	0.839	0.836	0.882	0.963	0.843	0.879	0.908	0.905
Level-3 (30%)	0.799	0.769	0.828	0.963	0.810	0.846	0.872	0.881
Level-4 (40%)	0.765	0.732	0.807	0.956	0.776	0.811	0.842	0.880
Level-5 (50%)	0.728	0.683	0.768	0.956	0.768	0.779	0.816	0.881
Average	0.8024	0.784	0.843	0.9602	0.8142	0.847	0.877	0.8904
(b) Metal-nut	AUC Score				F1 Score			
Poison Samples	PaDIM [47]	KDE Modeling-based	Probabilistic Modeling-based [60]	HIT + MiLF	PaDIM [47]	KDE Modeling-based	Probabilistic Modeling-based [60]	HIT + MiLF
Level-1 (10%)	0.948	0.737	0.871	0.955	0.939	0.90	0.947	0.968
Level-2 (20%)	0.865	0.713	0.802	0.918	0.940	0.898	0.942	0.941
Level-3 (30%)	0.862	0.719	0.777	0.906	0.940	0.90	0.938	0.941
Level-4 (40%)	0.820	0.732	0.736	0.889	0.947	0.906	0.938	0.946
Level-5 (50%)	0.820	0.732	0.727	0.859	0.930	0.906	0.929	0.936
Average	0.863	0.726	0.782	0.905	0.9392	0.902	0.938	0.946

## Data Availability

Publicly available datasets were analyzed in this study. The data can be found here: https://www.kaggle.com/datasets/ravirajsinh45/real-life-industrial-dataset-of-casting-product (accessed on 11 November 2021).
